# Patient Engagement in Health Research: Perspectives from Patient Participants

**DOI:** 10.3390/curroncol30030210

**Published:** 2023-02-26

**Authors:** Julie Easley, Richard Wassersug, Sharon Matthias, Margaret Tompson, Nancy D. Schneider, Mary Ann O’Brien, Bonnie Vick, Margaret Fitch

**Affiliations:** 1 Department of Medical Education, Horizon Health Network, Dr. Everett Chalmers Regional Hospital, 700 Priestman Street, Fredericton, NB E3B 5N5, Canada; 2 Department of Cellular & Physiological Sciences, University of British Columbia, Vancouver, BC V6T 1Z3, Canada; 3 Matthias Inc.: Connecting for Innovation and Advancing Societies, Edmonton, AB T5K 1S5, Canada; 4 Independent Researcher, Saskatoon, SK S7H 0A1, Canada; 5 St. Walburg Town Hall, St. Walburg, SK S0M 2T0, Canada; 6 Department of Family and Community Medicine, Temerty Faculty of Medicine, University of Toronto, Toronto, ON M5G 1V7, Canada; 7 Independent Researcher, St. Walburg, SK S0M 2T0, Canada; 8 Bloomberg Faculty of Nursing, University of Toronto, Toronto, ON M5T 1P8, Canada

**Keywords:** patient participation, community-based participatory research

## Abstract

Background and purpose: Over the past decade, patient engagement (PE) has emerged as an important way to help improve the relevance, quality, and impact of health research. However, there is limited consensus on how best to meaningfully engage patients in the research process. The goal of this article is to share our experiences and insights as members of a Patient Advisory Committee (PAC) on a large, multidisciplinary cancer research study that has spanned six years. We hope by sharing our reflections of the PAC experiences, we can highlight successes, challenges, and lessons learned to help guide PE in future health research. To the best of our knowledge, few publications describing PE experiences in health research teams have been written by patients, survivors, or family caregivers themselves. Methods: A qualitative approach was used to gather reflections from members of the Patient Advisory Committee regarding their experiences in participating in a research study over six years. Each member completed an online survey and engaged in a group discussion based on the emergent themes from the survey responses. Results: Our reflections about experiences as a PAC on a large, pan-Canadian research study include three overarching topics (1) what worked well; (2) areas for improvement; and (3) reflections on our overall contribution and impact. Overall, we found the experience positive and experienced personal satisfaction but there were areas where future improvements could be made. These areas include earlier engagement and training in the research process, more frequent communication between the patient committee and the research team, and on-going monitoring regarding the nature of the patient engagement. Conclusions: Engaging individuals who have experienced the types of events which are the focus of a research study can contribute to the overall relevance of the project. However, intentional efforts are necessary to ensure satisfactory involvement.

## 1. Introduction

Over the past decade, patient engagement (PE) has emerged as an important way to help improve the relevance, quality and impact of health research [1,2,3,4]. The Canadian Institutes of Health Research (CIHR) defines PE as the active and meaningful collaboration and involvement of patients in all parts of the research process [5]. This includes the overall governance of the research, setting priorities, conducting the research, and the translation and dissemination of research results [1,5,6,7].

PE in research can vary, from a perfunctory token presence to being an active partner in all aspects of the research [3,8,9]. Motivation for health researchers to include PE stems from the recognition that patients, survivors, family members or informal caregivers have lived experience expertise, which may extend the expertise of other research team members [3,9,10]. PE has also been deemed an important criterion for health research funding agencies [2,9,11,12]. However, despite recognition of the potential value of PE in research, there is limited consensus on how best to meaningfully engage patients in the research process [1,10,13,14].

The goal of this article is to share our experiences and reflections as members of a Patient Advisory Committee (PAC) on a large, multidisciplinary cancer research study that spanned six years (2013–2019). The Canadian Team to Improve Community-Based Cancer Care Along the Continuum (CanIMPACT) is a pan-Canadian team of researchers, health professionals, and knowledge users committed to improving care for cancer patients [15]. We hope by sharing our reflections of the PAC experiences on CanIMPACT, we can highlight successes, challenges faced, and lessons learned to help guide PE in future health research. All authors of this article are members of the PAC and participated in this reflective exercise to share our insights.

### 1.1. Background

A growing body of literature has highlighted the benefits of incorporating PE in research [1,3,10,16,17]. However, researchers attempting to include patients and family caregivers often face challenges [3,9,18]. PE in research can be messy; it often requires added time, resources, and expertise to help facilitate the engagement, which may not always be available [1,18,19]. It can also be challenging to manage the expectations of all those involved and define clear roles for the patients [1,16].

One particular challenge is recruiting patients who represent a diverse background yet still speak for the collective patient experience [14,17,19,20,21]. Research that involves intensive PE can be time consuming and may preclude participation by those without a lot of time or energy to invest [22]. This may lead to certain groups of patients being more likely to get involved than others and may limit the diversity of the patient voices represented [17]. Researchers may also be inclined to maintain relationships with patients already known to the research team to avoid recruitment challenges [14]. It is questionable whether these individuals would still provide perspective of the lay expertise after having gained experience and knowledge about research from past endeavours [17].

Systematic reviews of PE in research have shown that many efforts to engage patients are generally quite tokenistic and are frequently poorly reported [21,23]. Engaging patients and family caregivers can feel like ‘just a check mark’ to satisfy funders and not truly enriching the research process [4,9,14,22]. Certain types of research and methods also lend themselves more easily to PE than others. This may limit the level and types of PE and make it more challenging for researchers to find ways to meaningfully engage [9]. In general, early involvement by patients and caregivers as the research study is being planned facilitates relevant dialogue and engagement.

Several PE frameworks have been developed recently; however, there is no uniformly recognized model for effective PE with research projects being so varied [4,5,7,14,17,20,23]. Overall, there is consensus across the frameworks that finding what works best for each individual research project and having patients and family caregivers involved as part of the PE plan upfront are beneficial. Additionally, the engagement approach should be routinely evaluated to help ensure the patient voice is continually recognized and incorporated throughout the process in a way that enhances the research [1].

In Canada, the Canadian Institutes for Health Research (CIHR) launched its Strategy for Patient-Oriented Research (SPOR) in 2011 which helped make PE in health research a priority in Canada. Subsequently, CIHR released its Patient Engagement Framework in 2014 [2,5,12]. Around this time, the CIHR also launched a Community-Based Primary Health Care (CBPHC) initiative to help support and encourage interdisciplinary, cross-jurisdictional teams of researchers and decision makers in Canada. The goal was to conduct research focused on two key priority areas: access to care for vulnerable populations, and chronic disease prevention and management [24]. The funding application encouraged teams to outline their patient advisory process to ensure patient feedback and input on all aspects of the research [2].

### 1.2. The Canadian Team to Improve Community-Based Cancer Care along the Continuum (CanIMPACT)

Our PAC was involved with CanIMPACT, one of 12 CBPHC Innovation Teams to receive funding through the CIHR initiative. The overall goal of CanIMPACT was to help elucidate gaps in care and develop strategies to enhance the capacity of primary care for cancer patients and improve integration between primary care and cancer specialist care along the cancer care continuum [15]. The researcher team was comprised of primary care physicians, nurses, oncology specialist physicians, researchers, knowledge users, patients, and caregivers from across Canada.

**Figure 1:**
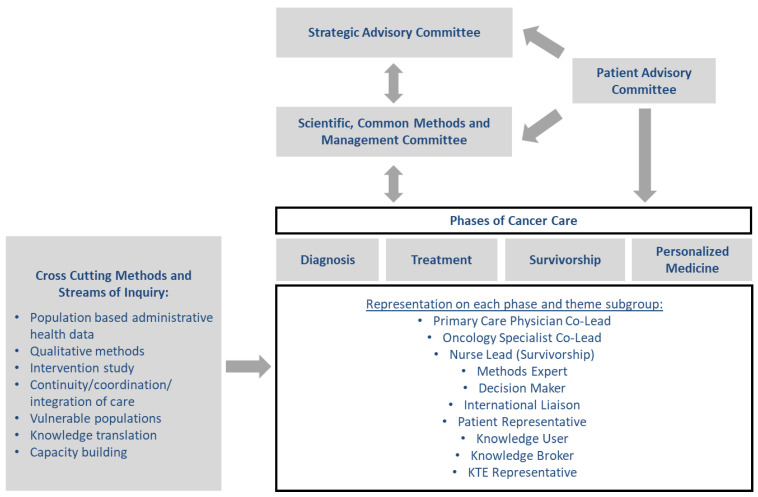
CanIMPACT Research Program Organizational Structure [25].

Using a mixed methods approach, the activities of the CanIMPACT team were divided into 2 phases. Phase 1 included foundational research using four sub-groups: (1) population-based administrative health databases; (2) qualitative interviews with family physicians, specialists, and patients; (3) an environmental scan and systematic review of existing initiatives; and (4) an exploration of issues related to personalized medicine. The results of Phase 1 were presented at a consultative workshop with key stakeholders that gave direction for Phase 2, which was a pragmatic, randomized controlled trial to pilot test an intervention to improve the co-ordination of cancer care between oncologists and family physicians [15]. The CanIMPACT Patient Advisory Committee (PAC) served as an overarching committee, as well as having individual patient representatives integrated into each of the research subgroups (see Figure 1).

### 1.3. CanIMPACT Patient Advisory Committee

Our PAC was comprised of patients and family members, who had lived through a cancer experience. The stated purpose of the PAC within CanIMPACT was to ensure patient and family perspectives were brought to the forefront of all CanIMPACT activities. Three research team members were also a part of our PAC, each with their own lived experience with cancer or as caregivers. The group was co-chaired by a patient/family representative and a researcher. Administrative support was provided to the PAC to help facilitate meetings and keep the PAC updated and connected to the rest of the research team. One PAC member also worked as a research assistant during Phase 1 of the research study and helped with qualitative data collection and analysis. Most PAC members were recruited through networks of research team members or through other PAC members.

Having been engaged with health research as patients/survivors and caregivers over six years, we developed insights about the engagement process which we believe could interest a broad range of stakeholders. We believe there are lessons we can share that contribute to the evolving body of evidence and practices regarding how to successfully engage patient and family caregivers in research endeavours. To the best of our knowledge, few publications describing PE experiences on health research teams have been written by the patient/families themselves.

## 2. Methods

To capture our experiences and reflections as a PAC, we used a qualitative approach. We involved members in reflecting about their PAC experiences by using a systematic framework of questions distributed through an online survey, followed by group discussion based on the collected perspectives to help gather our retrospective thoughts and insights. This allowed us to review and evaluate our experiences and reflections about the impact of our involvement as a PAC over the past six years with CanIMPACT. Ethics approval was granted by the Horizon Health Network Research Ethics Board, New Brunswick, Canada (File # 100776).

### Data Collection and Analysis

One PAC member (JE) with research training and experience in qualitative methods took the lead, inviting all past and current members involved on the PAC to participate in a reflective exercise and contribute to this article. After preliminary discussions as a group, the leader e-mailed a list of questions to the PAC members to help guide their reflections on perceived successes, challenges, and areas for future improvement. (See Table 1). This allowed members to initially reflect on their experiences as individuals, before discussing overall insights as a group. Each PAC member had opportunity to share their thoughts and experiences either by e-mail or phone with the leader. Once all data were collected, e-mails and notes from phone interviews were collated and analyzed by the leader using thematic analysis to identify common themes [26]. These common themes were presented to the PAC members to facilitate the group discussion. Over a Zoom call, the group reviewed and discussed the emergent themes from the individual reflections and agreed on the messages we felt were most pertinent to share.

## 3. Results

### 3.1. Participants

All current members of the PAC (n = 8) agreed to participate; one former member declined, and one former member did not respond. The PAC included both patients (a range of ages and disease sites) and caregivers from across Canada, including the Atlantic provinces, Ontario, the Prairies, and the West Coast.

### 3.2. Summary of Our Reflections

Our reflections about experiences as a PAC on a large, pan-Canadian cancer research study fit under three headings: (1) successes/what worked well; (2) challenges/areas for improvement; and (3) reflections on our overall contribution and impact.

### 3.3. Successes/What Worked Well

#### 3.3.1. Great Working Relationships and Treated with Respect

One of the biggest strengths for PAC members was having great working relationships and camaraderie among members of the PAC and with the investigative research team. Despite our different cancer experiences and backgrounds, we functioned well as a group and were able to come together to speak for the collective patient experience, not just as individuals. We were consistently treated with respect by the investigative research team members, and all felt that team members were willing to listen to our input when the opportunity was presented. We feel we succeeded in increasing awareness about patient experience and recognition of the value of the patient voice in research because of this open and respectful research environment.

#### 3.3.2. Administrative Support and PAC Co-Chair Roles with Research Team Member and Patient Representative

Two administrative and structural factors were key contributors that helped us function well as a PAC. One was the administrative support provided by the Research Project Coordinator. Having this support helped overcome the challenges of coordinating PAC meetings with members from across Canada in different time zones. The coordinator also helped with travel and logistics to attend team meetings and provided a vital link to the overall research team, which helped keep us updated on the research. Without this support, we would have been limited in our ability to work together as a PAC and interact with investigators. Another key contributor to our success as a PAC was having research team members on our committee, with one serving as co-chair together with a patient representative. This provided another vital link to keep us connected to the overall research team. This leadership helped provide insight and answers to questions that arose as the study progressed and helped to prompt the team where there were ways for the PAC to contribute. It helped integrate the PAC with the rest of the research team and provided consistency as project staffing changed and corporate memory dissolved.

#### 3.3.3. Patient Experience Synthesis Map

The PAC helped develop a Patient Experience Synthesis Map, a visual illustration of what can happen to individuals diagnosed with cancer. This activity was perceived by most members of the PAC to have been the most noticeable contribution to the project. Synthesis mapping is a unique knowledge translation tool and technique used for visual interpretation of research evidence [27]. CanIMPACT worked with The Strategic Innovation Lab at the Ontario College of Art and Design University (OCAD) to develop a synthesis map to communicate salient issues in the Canadian cancer system based on research evidence.

As the Cancer Care Pathways Synthesis Map was being developed by the research team, the PAC had opportunity to provide feedback and collectively pointed out that we felt there was not enough of the patient experience represented in the clinical map. As one PAC member describes, “*we simply told them that we do not see ourselves in this health system map”.* As a result, a second synthesis map was developed in consultation with the PAC providing a visual narrative of patient experience with the cancer care system. This map illustrated the patient journey through two fictional characters and how the cancer experience can impact their lives in various ways. The map attempted to depict the wholistic nature of the experience and the impact of social determinants of health. Most PAC members felt that this was a meaningful moment in the research when our voices were heard, and changes were made to the map to better reflect the patient experience. It was also an activity during which PAC members felt most engaged or active in the research project. Members observed this mapping exercise fostered discussion on patient experience with the whole research team and contributed to decisions about the approach in Phase 2 of the study. The map itself was a tool used in communicating about patient experience as the research moved forward. More information on the CanIMPACT synthesis maps can be found in detail elsewhere [27].

### 3.4. Challenges/Areas for Improvement

#### 3.4.1. Timing of Engagement

All PAC members agreed that engagement from the initial planning stages would have been beneficial. Recruitment of PAC members did not begin until after funding had been secured and the research program was fully planned and already underway. However, recruitment was challenging, and by the time the PAC met for the first time most Phase 1 research design decisions had already been made. As one PAC member described, *“it was like being asked to board a train that was already moving without having any say on the destination or route”.* We recognized this is a common shortcoming of many research studies when engaging patients and family caregivers and how it can be difficult to assemble a patient advisory group before receiving funding for resources to facilitate that engagement. However, with much of the research design already established as part of the funding application, it can be difficult to know where input from the PAC members will be welcomed and utilized. Ideally, funding and resources are needed for research teams to support PE in the development stages of a research project.

#### 3.4.2. Training/Guidelines Needed

Although it was recognized that PE input was important when CanIMPACT first started, there were few resources or guidelines available at that time on how best to meaningfully engage patients. There was a learning curve for both PAC members and the investigative team regarding how to integrate the patient voice into the research. Initially, there was a struggle for both PAC members and the investigative team to find the best approaches for PE. For the PAC, we needed more guidance, not only on how to work with the investigative team, but also on how to work together as a PAC. It would have been helpful, for example, to have more discussion with the investigative team on the role and expectations of the PAC. Early in the project, we often had to develop ideas on how to incorporate ourselves in the work and put suggestions about engagement forward, instead of these types of ideas coming from the investigative team. We had to learn quickly how to be proactive.

#### 3.4.3. Tokenism

There were moments in the beginning of the PAC where we had feelings that a token checkmark for PE had been made. It was challenging to get the research team to see PAC members as partners and not just present “because funders want it”. Some PAC members felt sidelined in the early stages and not as integral part of the project with perspectives which could enrich that part of the project. As the project progressed and more interaction occurred, PAC members felt their input was increasingly valued. However, there was a learning curve for all and continued need for reminders regarding ways the PAC could contribute as the research study progressed.

#### 3.4.4. More Communication and Opportunities to Interact with the Research Team

A great deal was accomplished during face-to-face team meetings to help establish relationships and connection with other PAC members and with the rest of the research team. Frequent face-to-face meetings were not feasible financially, so various project teams had to rely on teleconferences to connect. Teleconferences were a great way to keep a widely dispersed group connected between in-person meetings, but it did not allow much opportunity to further develop relationships and connections with other project teams. As the PAC, we functioned primarily on our own with our own meeting schedule, periodic updates about investigative team activities, and occasional opportunities to participate in the research process. There were times when we felt that we were being primarily informed rather than actively engaged. More opportunities to interact with the whole research team would potentially have helped us to feel more engaged, and contributory to the research.

#### 3.4.5. PAC Recruitment Challenges and Lack of Diversity

One area that proved to be challenging was the recruitment of PAC members and ensuring a diverse representation of patients and family members. Despite recruitment efforts, most PAC members were recruited through previous connections with research team members or with other PAC members. It was particularly challenging as a pan-Canadian team to find members who were willing to commit for 6 years and were able to travel for meetings. Although the PAC was geographically diverse (from five provinces including rural and urban settings), the group lacked diversity in gender (only one male), ethnicity (all Caucasian), language (all Anglophone) and reflected a higher level of post-secondary and socio-economic background than might be found in a typical cancer clinic. Although we did our best to be inclusive of all cancer patients when describing patient experiences to research team members, there were undoubtedly patient voices missing from the discussions who may have represented other viewpoints.

#### 3.4.6. Need for Midpoint Evaluation of the PAC to Find Ways to Improve Engagement

PAC members agreed it would have been beneficial to have engaged in process evaluation at the midpoint of the research project to review PAC activities and look for opportunities to improve engagement. This type of activity could have helped to obtain perspectives from the research leaders of the four subgroups and the PAC members and might have led to changes to enhance the PAC role.

### 3.5. Reflections on Our Overall Contribution and Impact

All PAC members agreed there were meaningful moments of engagement for us throughout the study. For example, helping with semantics and wording on a document to make it more patient-centered; reviewing questions for qualitative interviews; and the development of the patient synthesis map were seen as meaningful. We did note that some research sub-groups were better than others at soliciting PAC input, although we also recognized that some types of research activities lend themselves more easily to PE than others. The personalized medicine sub-group did particularly well at creating space for PE throughout the span of their work, and individual PAC members involved with this group felt they contributed in a substantial way.

However, feelings were mixed on whether we truly made a meaningful contribution to the overall CanIMPACT initiative and the actual research study. We all agreed that over the span of the study there was better recognition of the value of the patient perspective and perhaps a change in attitude of the investigative team regarding PE in research. A willingness to listen and attempts to engage did evolve. Yet, a few PAC members felt that we were not particularly challenged with any task that might have led to us influencing research design or outcomes in any substantial way. This is not a criticism but more of a realistic assessment of what was expected of us and what we delivered. It epitomizes the research world perspective at that time regarding PE. As one PAC member described,

“*We were informed and engaged as requested. We were approached respectfully and responded in kind. We met our obligation of affirming that the program had invited and engaged the patient population. But there were no crucial questions brought to our attention nor were we presented problems or questions that would have required real intellectual deliberation from a panel of cancer patients*.”

Another PAC member noted, *“Our level of engagement was good at the time for what was known about PE at the beginning of the project. But lots has changed and improved over the last six years, so things may be a lot different if we were starting out now.”* Overall, the entire PAC agreed it was a very positive experience to be part of the CanIMPACT initiative and our involvement held meaning and importance to all of us personally, regardless of the research outcomes.

## 4. Discussion

The goal of this exercise was to reflect on our engagement experiences as patients and family caregivers and share our insights about working together on a multidisciplinary, pan-Canadian research study that spanned six years. We identified various strengths and areas for improvement in hopes of enriching PE activities for future research teams. Our reflections lead to recommendations in three areas: communication and connection, early engagement and training, and evaluation of PE opportunities throughout the research program.

### 4.1. Communication and Connection

Communication has been emphasized by many as a key to effective PE [1,2,28,29]. Effective communication requires open dialogue and achieving mutual trust, respect, integrity and transparency that allows patients timely input to research activities [9,10,28,30]. Establishing connections and building relationships between PAC members and investigative team members is essential in helping patients feel empowered and confident enough to share their voice [7]. This connection also helps build a bridge for research team members to feel more comfortable reaching out to PAC members and helps foster a two-way dialogue. For our PAC, having opportunity to interact with the research team in person, whether informally or as part of a research activity, helped build these relationships. Although not always feasible due to lack of resources and being geographically dispersed, face-to-face meetings with both the PAC and the full research team helped us feel part of the research program. Having an individual dedicated to administrative tasks and communications was also helpful, as well as having research team members on our PAC.

### 4.2. Early Engagement and Training

Including patients early in the research process and providing appropriate training for both PAC and research team members are frequently cited as essential components to fostering meaningful engagement [1,4,10,16]. Orientating patients to the research process and training them to actively engage and work within a team has been shown to enhance PE [1,16]. It is important to note that the goal of providing training to patient representatives is not necessarily to turn them into researchers. Rather it is to give them tools to better understand the research process and feel more confident participating in discussions [10].

Conversely, training researchers how to effectively communicate and engage with patients has been shown to be equally important in fostering participation by both parties [16]. Ideally, the opportunity for co-learning would be another way to further build relationships and establish rapport between all team members [16]. Most importantly, a framework or engagement plan should be established prior to starting the research itself [1,10].

### 4.3. Evaluation Throughout

Engaging in ongoing evaluation of PE is essential [1,9,16]. Incorporating an evaluation process throughout the research initiative will help ensure the engagement experience is meaningful and positive for all involved [9,10,31]. It also helps ensure patients and family caregivers remain active and informed, with opportunity to adjust and adapt engagement strategies based on feedback obtained by both patients and researchers [9]. Without adequate evaluation, it is difficult to assess near, intermediate and long-term outcomes and learn from current practices [1,31].

## 5. Limitations

To the best of our knowledge, few publications describing PE experiences in health research teams have been written by patients, survivors, or family caregivers themselves. Although this is a strength of our approach, it also lends itself to potential bias. We are uniquely positioned as both the participants and the authors in this case, sharing a firsthand account of our own experiences and insights as a PAC. We used a qualitative approach to engage in reflective discussions as a group; however, it does not meet all the standard criteria of more traditional qualitative methodologies. Our reflections are of our own experiences and may not be transferable to other studies or teams. However, we hope others may benefit from our shared insights when developing strategies for PE in future health research. 

## 6. Conclusions

Defining success and what constitutes meaningful contribution in PE depends on the goals and expectations of both the PAC and research team. Allowing space in early stages of a research endeavour to discuss and define PE goals and expectations together as a team is an essential step in building relationships, trust, and mutual respect, and fostering open dialogue. Although there is a lack of consensus on how best to engage patients, there is a growing body of literature that explores tools and shared insights to help research teams define these roles, and thus manage expectations. Hopefully our observations can contribute to further develop robust PE strategies for future research initiatives. As a PAC, we have been uniquely positioned at a time when PE was emerging in health research, and we have been able to watch it grow and evolve over the past six years. Where we started is not where we are now in terms of PE, and we hope that by sharing our reflections and insights others may learn from our experiences.

## Figures and Tables

**Table 1:** Questions for PAC Member Reflective Exercise.

1. How were you recruited to join the CanIMPACT PAC? What made you want to join? What made you want to continue to be involved over the past 6 years?
2. In terms of the study itself and the PAC involvement, what do you think has worked well? What are some of the strengths and successes of the PAC and the CanIMPACT study?
3. What do you feel could have been (or could still be) improved? What have been some of the challenges faced by the PAC?
4. Do you feel that the PAC has made a meaningful contribution to the CanIMPACT study? If yes, any examples? If no, why do you feel this way?
5. Do you feel you have benefitted from being involved with the CanIMPACT Study and being a part of the PAC? What will you take away from the experience at the completion of the study?
6. Are you involved with other PACs? If so, how does this one differ? Would you join another PAC based on your experience with CanIMPACT?
7. Is there anything that could have been done to improve your overall experience as a member of the PAC?
8. Any other comments, reflections or lessons learned along the way?

## Data Availability

The original contributions presented in the study are included in the article, and further inquiries can be directed to the corresponding author at julie.easley@horizonnb.ca.
